# COVID-19 Vaccination Intention and Vaccine Hesitancy among Patients with Autoimmune and Autoinflammatory Rheumatological Diseases: A Survey

**DOI:** 10.1155/2022/5931506

**Published:** 2022-01-31

**Authors:** Samar Tharwat, Haidy Adel Abdelsalam, Adel Abdelsalam, Mohammed Kamal Nassar

**Affiliations:** ^1^Rheumatology & Immunology Unit, Department of Internal Medicine, Faculty of Medicine, Mansoura University, Mansoura, Egypt; ^2^Mansoura Manchester Programme for Medical Education, Faculty of Medicine, Mansoura University, Mansoura, Egypt; ^3^Mansoura Nephrology & Dialysis Unit (MNDU), Department of Internal Medicine, Faculty of Medicine, Mansoura University, Mansoura, Egypt

## Abstract

**Background:**

Coronavirus disease 2019 (COVID-19) vaccine hesitancy or refusal has arisen as a major global public health concern. The aim of this study was to address the attitudes of patients with autoimmune and autoinflammatory rheumatological diseases (AIIRDs) about COVID-19 vaccination and investigate the factors that influence their decision.

**Methods:**

This interview-based cross-sectional study was carried out on AIIRD patients at the period between 15 August and 15 September 2021. The questionnaire included socioeconomic data, intention to receive COVID-19 vaccine, AIIRD subtype, disease duration, associated comorbidities, history of COVID-19, beliefs and attitudes towards COVID-19, and conventional vaccination in general and COVID-19 vaccine in particular, in addition to COVID-19 vaccination status.

**Results:**

A total of 206 AIIRD patients were included, with a mean age of 37.61 years (SD = 10.67), and 84% were females. The percentage of vaccine acceptance was 70.39%, while only 16.02% were hesitant and 13.59% were resistant to COVID-19 vaccination. About one-fourth of patients reported getting infected with COVID-19. Of them, 15.1% were hospitalized and 5.7% were admitted at the intensive care unit (ICU). Most of the AIIRD patients (77.2%) believed that they are at a higher risk of getting COVID-19. The main motivation for vaccine acceptance was the fear of being infected (41.4%). About 40% of vaccine nonacceptants fear about the serious side effects of COVID-19 vaccine.

**Conclusion:**

There is a high acceptability rate of COVID-19 vaccination among AIIRD patients. Public health workers and policymakers must emphasize efficient COVID-19 vaccine acceptance messaging for all AIIRD patients.

## 1. Background

Coronavirus disease 2019, which is known as COVID-19, is caused by a new coronavirus termed severe acute respiratory syndrome coronavirus 2 (SARS-CoV-2) [1]. COVID-19 was declared a global pandemic by the World Health Organization (WHO) on March 11, 2020 [2]. The COVID-19 pandemic is now without a doubt not only a global health emergency but also a severe global economic downturn, as the death rate increases and economies are intentionally shut down [3].

Autoimmune and autoinflammatory rheumatological diseases (AIIRDs) are a diverse group of disorders that typically affect the musculoskeletal system and connective tissue [4]. In comparison to the general population, the prevalence of COVID-19 among AIIRD patients was shown to be higher [5, 6]. Preventive lifestyle measures, such as social distancing and the use of personal protective equipment, have been proposed as effective techniques to minimize the rate of SARS-CoV-2 infection in AIIRD patients [7].

In the general population, as well as in individuals with AIIRDs, vaccination is an important and effective tool in preventing COVID-19. Additionally, people who are vaccinated against this infection are less likely to develop severe disease [8]. Rapid development of safe and efficient COVID-19 vaccinations has the potential to halt the global COVID-19 epidemic.

However, vaccine hesitancy, or the unwillingness or refusal to be vaccinated, has arisen as a major global public health concern, particularly because it may hinder the ability to achieve herd immunity status [9–11].

AIIRD patients are considered a priority group for COVID-19 vaccination [12]. However, little is known about their beliefs and awareness of COVID-19 vaccination. Vaccine hesitancy or resistance in those with AIIRDs can be a roadblock [13].

As a result, it is important to know AIIRD patients' attitudes towards COVID-19 vaccination. It would be useful to know this in order to design targeted strategies to improve acceptability and overcome the hesitancy problem. To the best of our knowledge, vaccine acceptability among AIIRD patients, as well as actual translation into vaccine uptake, has not yet been assessed in Egypt.

As a result, the aim of this study was to determine the level of intention to get vaccinated against COVID-19 among Egyptian patients with AIIRDs, as well as to estimate their knowledge and beliefs towards COVID-19 vaccination.

## 2. Methods

### 2.1. Study Design and Settings

This interview-based cross-sectional study was carried out at the period from 15 August to 15 September at a rheumatology and immunology unit in Lower Egypt. The work complies with the ethical standards of the 1995 Helsinki Declaration and was approved by the institutional review board of Mansoura University (Approval No: R.21.10.1468). The study included consecutive patients with the diagnosis of AIIRDs visiting the outpatient clinic. Patients aged less than 18 years or who had degenerative joint diseases unrelated to AIIRDs and neurological or psychological disabilities were excluded from the start. All participants were provided with detailed information about the study, and informed written consent was obtained from them.

### 2.2. The Questionnaire

The questionnaire was prepared and then revised and refined. After being translated, it was distributed to the patients in Arabic. A pilot study was conducted to validate the questionnaire, and 5 rheumatology staff members reviewed the correctness of the translation, questionnaire design, content, words, comprehension, and ease of completion. The questions were made in an attempt to be as simple as possible, with the answers limited to yes/no (closed-ended questions) except for the questions about assessment of beliefs about conventional vaccination.

The survey included the following areas: socioeconomic data, intention to receive COVID-19 vaccine, AIIRD subtype, disease duration, associated comorbidities, history of COVID-19 and its sequelae, and therapeutic history.

**Figure 1:**
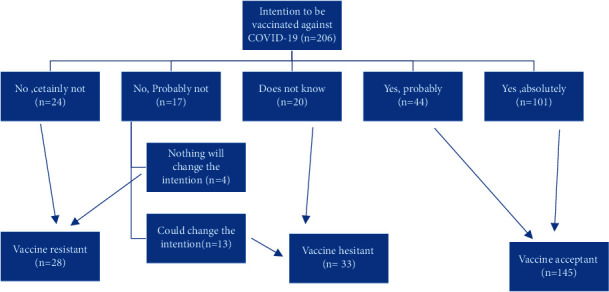
Classification of AIIRD patients according to the intention to receive COVID-19 vaccine (*n* = 206).

To characterize the distribution of the characteristics, a descriptive analysis was used. The vaccine acceptant (VA) group included AIIRD patients who answered “Yes, absolutely” or “Yes, probably” to the COVID-19 vaccine intention question; the vaccine hesitant (VH) group included those who answered “No, probably not” or “I do not know” to the COVID-19 vaccine intention question; and the vaccine resistant (VR) group included those who answered “No, certainly not” or “No, probably not” to the COVID-19 vaccine intention question (Figure 1).

The questionnaire also included questions about perception, beliefs, and attitudes towards COVID-19, conventional vaccination in general and COVID-19 vaccine in particular, and source of information about COVID-19 vaccine. The document also explored the perceived motivators of COVID‐19 vaccination among the VA group and barriers of COVID-19 vaccine among VH and VR groups.

The final part concerned with COVID-19 vaccination. Participants were asked about their COVID-19 vaccination status before proceeding to the rest of the questions. If the participant had been vaccinated, we asked about the type of vaccine and then we asked about side effects separately, including anaphylaxis, skin rash, fever or chills, widespread muscle or joint pain, fatigue or sleepiness, headache, nausea, vomiting, poor appetite, and chest pain or palpitations.

Patients were contacted (in person), and the questionnaire was explained to them, along with trade name examples of generic pharmaceutical names. Patients with a poor level of education were guided on how to fill out the questionnaire. It was created to take about 5 to 10 minutes to be completed. The responses were recorded and transferred into an excel spreadsheet.

### 2.3. Statistical Analysis

Quantitative data were reported as means ± standard deviation (SD) for parametric variables or median (min-max) for nonparametric variables, while qualitative data were described as percentages and numbers. The Shapiro–Wilk test was employed to determine the normality of the variable distribution. The one-way ANOVA test was used for normally distributed data, while the Kruskal–Wallis test was used for nonnormally distributed variables when comparing VA, VH, and VR groups. For comparing qualitative variables, the chi-square and Fisher exact tests were used. *P* value <0.05 was considered significant.

## 3. Results

### 3.1. Demographic Characteristics and Clinical Data

A total of 206 AIIRD patients were included in the study (Table 1), with a mean age of 37.61 years (SD = 10.67), 84% were females, and most of them (88.3%) were educated. The percentage of vaccine acceptance was high (70.39%), while only 16.02% of the participants were hesitant and 13.59% were resistant to COVID-19 vaccination.

As shown in Table 2, the most encountered AIIRDs were systemic lupus erythematosus (SLE) (41.3%), rheumatoid arthritis (RA) (26.2%), systemic sclerosis (SSc) (14.1%), and Behçet's disease (BD) (10.7%). The median (min-max) disease duration of AIIRD was 5 (0.5–35) years. About one-fourth of patients with AIIRDs reported that they had suspected or confirmed COVID-19 in the previous 12 months. Of them, 15.1% were hospitalized and 5.7% were admitted at the intensive care unit (ICU).

### 3.2. Perception, Beliefs, and Attitudes and Sources of Information

As illustrated in Table 3, most of the AIIRD patients (77.2%) believed that they are at a higher risk of getting COVID-19 infection due to AIIRD. Even though, most of them thought that they are more susceptible to severe COVID-19 and COVID-19 vaccine adverse events due to AIIRD (83.5% and 75.2%, respectively). Confidence in the efficacy, security, usefulness, and estimated knowledge of conventional vaccines (excluding COVID-19 vaccines) was 7/10, 7/10, 7/10, and 6/10, respectively. Of the patients who accepted the vaccine, 89.7% were confident of its importance and 90% considered it essential for everyone in the community. However, only 30% of those who were vaccine resistant admitted its importance.

**Figure 2:**
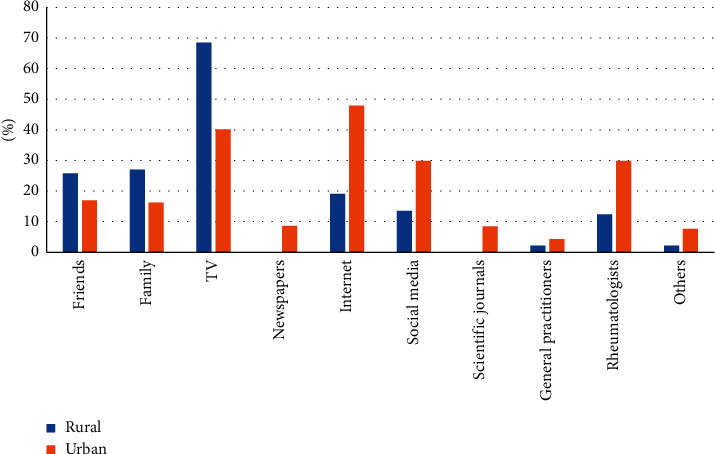
Sources of information about COVID-19 vaccine among the AIIRD population according to the residence (rural versus urban) (*n* = 206).

Concerning the sources of information about COVID-19 vaccine, television (TV) represented the major one for AIIRD patients from rural areas (70%), while the Internet attained the highest percentage (nearly 50%) in urban areas. Rheumatologists were the source of knowledge in 30% in AIIRD patients from urban areas and 12% in those from rural areas. General practitioners rated the least one with the same ratio in both regions as presented in Figure 2.

### 3.3. The Perceived Motivators and Barriers of COVID-19 Vaccination

**Figure 3:**
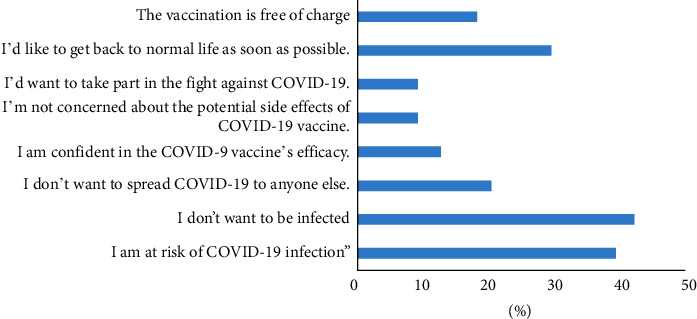
The motivators of COVID‐19 vaccination among the COVID-19 vaccine acceptant group (*n* = 145).

As illustrated in Figure 3, the main motivations for vaccine acceptance were the fear of being infected (41.4%), the perception of high risk of COVID-19 (38.6%), and the wish to return to normal life as soon as possible (29%).

**Figure 4:**
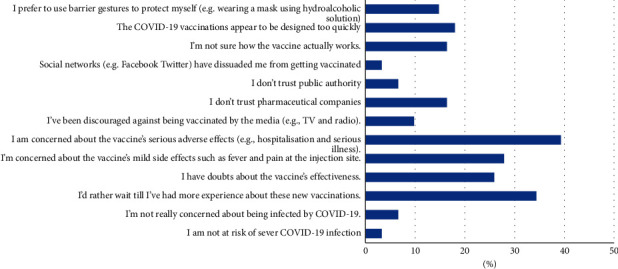
Barriers of COVID-19 vaccination among the COVID-19 vaccine hesitant and resistant groups (*n* = 61).

Figure 4 demonstrates the barriers to COVID-19 vaccination acceptability in VH and VA groups. About 40% fear about the serious side effects of COVID-19 vaccine. Approximately one-third (34%) preferred to wait until they have more experience about these new vaccines, while 28% were afraid of mild side effects and 26% doubted the efficacy of the COVID-19 vaccines.

### 3.4. List of Types and Adverse Effects of Received COVID-19 Vaccines

Among the studied 206 AIIRD population, 37(17.96%) patients have received COVID-19 vaccine. Sinopharm and AstraZeneca were the most common used vaccines (29.7% and 24.3%, respectively). The most common postvaccination symptoms were widespread muscle and/or joint pain (40.5%) and fever or chills (32.4%), followed by fatigue and sleepiness (16.2%). The less likely appeared symptoms were mouth dryness (2.7%) and diarrhea (2.7%). Two AIIRD patients reported flare of the existing AIIRD within few days after COVID-19 vaccination (Table 4).

## 4. Discussion

Nowadays, there is a respectable campaign all over the world to battle against the novel COVID-19 pandemic [14, 15]. As known, vaccination is the most efficient way to eradicate infection [16]. COVID-19 vaccine received different attitudes regarding its acceptance [17]. AIIRD patients have concerns about COVID-19 vaccination [18].

To the best of our knowledge, our study is the first one to determine the attitudes and beliefs towards COVID-19 vaccination among Egyptian patients with AIIRDs. The sample group was divided between vaccine acceptancy, hesitancy, and resistance. This finding illustrates the topic's dilemma in AIIRD patients, where half of them were willing to get the vaccine. Holding positive beliefs about conventional vaccination in general and COVID-19 vaccine in particular is the most important association of vaccination. The most common sources of information in rural and urban areas were TV and the Internet, respectively, while rheumatologists were the source of knowledge in only 22% of AIIRD patients. The fear of being infected was the main motivation of COVID-19 vaccination acceptance, while fear about serious side effects of the vaccine was the most hindering factor against vaccination.

AIIRD patients have an increased burden of infections due to underlying autoimmune disease [19], comorbidities, and immunosuppressive therapy [20]. Meanwhile, patients with AIIRDs do not appear to be at an elevated risk of SARS-CoV-2 infection in comparison to the general population [21]. Also, pediatric patients with AIIRDs and those receiving biologics may not be at increased risk of neither COVID-19 nor its serious complications [22, 23].

In this study, about one-fourth of patients with AIIRDs reported that they had a history of suspected or confirmed COVID-19 in the previous 12 months. Of them, 15.1% were hospitalized and 5.7% admitted at the intensive care unit (ICU). In AIIRD patients, the overall risk of hospitalization and death related to COVID-19 was not or just marginally elevated [6, 24, 25]. It is, however, critical to maintain optimum AIIRD care and take preventive measures such as vaccination to limit the risk and severity of COVID-19 [26].The prevalence of severe COVID-19 in patients with AIIRDs is still unclear [27]. Pablos et al. stated that AIIRD patients are susceptible to more severe COVID-19 that necessities hospitalizations [28].Certain immunomodulatory medications have been suggested to have a possible preventative or therapeutic effect in these patients [29]. Understanding the risk of COVID-19 in AIIRD patients is crucial, and there is still a number of unanswered questions. It is unknown how SARS-CoV-2 reinfection affects AIIRD patients.

Our study shows that, in August and September 2021, more than half of the AIIRD patients were VA, 16% were VH, and 14% were VR. A similar pattern of results was obtained in another study from Rome where acceptability in AIIRD patients was 54.9% [17]. Also, this is consistent with what has been found in a previous study carried out on 1266 patients with AIIRDs and found that 686 (54·2%) were willing to get vaccinated against COVID-19 and 408 (32·2%) were uncertain, while 172 (13·6%) were unwilling to get vaccinated [8]. In this context, we emphasize the necessity of increasing patient education towards COVID-19 vaccination.

Additionally, few studies found that males were more willing to get vaccinated than females [8, 30, 31]. However, no such difference was found in our study. This finding may be as a result of the female predominance of our study which is probably due to epidemiological facts of AIIRDs. Also, willingness to get COVID-19 vaccine was not associated with comorbidities or immunosuppressive drugs in our cohort.

During the last few years, evidence about the safety, immunogenicity, and efficacy of vaccination in AIIRD patients has markedly grown [20].The concept of vaccination is refused by nonnegligible numbers of the population [32]. Barello S. et al. suggested that one of the factors of uncertainty is the lack of sense of responsibility towards society and losing the role of becoming an active arm in the eradication of the pandemic [33]. It is uncertain how AIIRDs and immunosuppressive medications will affect AIIRD patients' immunological response to the COVID-19 vaccine because AIIRD patients were not routinely included in vaccination trials. As a result, uncertainties about the efficacy and safety of COVID-19 vaccinations in AIIRD patients may cause clinicians and patients to be hesitant to administer COVID-19 vaccine [34, 35]. However, in a recently published study, COVID-19 vaccine in patients with AIIRD was found to have reduced but acceptable short-term immunogenicity [36]. Also, COVID-19 vaccination resulted in an adequate immunogenic response with a tolerable safety profile in the majority of patients with AIIRDs, according to another study conducted on 686 AIIRD patients and 121 controls [37].

Healthcare providers including rheumatologists need to customize their messages strategically based on the intent to get vaccinated; in low-intention groups, emphasizing the vaccination's safety may be more effective, whereas in high-accessibility and high-intention groups, emphasizing practical issues such as vaccine cost and logistics may be more beneficial [38].

In this study, about 80% of vaccine nonacceptant patients have the misconception that they are at a risk of developing more adverse effects of the vaccine. Boekel et al. stated that COVID-19 vaccines are well tolerated by patients with autoimmune diseases [31].

Almost all rheumatologists recommend the COVID-19 vaccine for their AIIRD patients even without lowering immunomodulatory drugs [39]. Strong agreement was reached on the consensus that COVID-19 vaccination should not be delayed for patients using hydroxychloroquine, sulfasalazine, leflunomide, apremilast, or IV immunoglobulin. For the majority of the remaining immunomodulatory treatments evaluated, a similar recommendation was reached with moderate consensus [40].

So, rheumatologists can positively influence vaccine acceptance in AIIRD patients through counselling about vaccine efficacy and safety concerns [40]. When vaccination against COVID-19 was advised by a physician, the willingness to get vaccinated increased, and the specialist physician was the most trusted healthcare provider [8].

From the results, it is clear that vaccine acceptancy is strongly associated with positive beliefs and attitudes towards vaccination in general and COVID-19 vaccination in particular. This is in agreement with the VAccinations against COVID-19 (VAXICOV) study [8].Vaccine uptake has been linked to a lack of vaccine information and poor attitudes regarding vaccination in general [41]. To overcome COVID-19 vaccination skepticism and, hopefully, put a stop to the epidemic, rheumatologists and AIIRD patients urgently require coordinated national and worldwide local awareness campaigns [12].

Our results cast a new light on the sources of information about COVID-19 vaccine in AIIRD patients. In this study, while TV and the Internet were the main sources of information, rheumatologists were considered the source of COVID-19 vaccine information in only one-fifth of AIIRD patients. It is notable that concerns have been raised about patient misinformation about the COVID-19 pandemic and vaccination safety [42]. So, rheumatologists should play a pivotal role in implementing vaccination education programmes [40].

Our results demonstrated that the main motivations for vaccine acceptance were the fear of being infected (41.4%) and the perception of high risk of COVID-19 (38.6%). A similar conclusion was reached by the VAXICOV study in which patients with AIIRDs expressed their willingness to get vaccinated against COVID-19 in order to protect themselves, their families, and the general population [8].

From the results, it is clear that the fear about the serious side effects of COVID-19 vaccine and the doubts about vaccination efficacy are the main barriers to COVID-19 vaccination acceptability, similar to those observed in prior studies [12, 13, 17, 30].

In those who received COVID-19 vaccine, only 2 patients experienced flare of their autoimmune disease few days shortly after COVID-19 vaccination. In a study carried out on 264 patients with AIIRDs who were assessed 4–6 weeks after receiving the second dose of COVID-19 vaccine, only minor side effects were reported and no apparent impact on AIIRD activity was noted [43]. So, further studies are warranted to declare this point.

This study was not without its limitations. The main limitation of this study is the absence of data about nonrespondents. The relatively small number of the participants from a single rheumatology center and the lack of data about healthy individuals may be another limitation. Furthermore, the cross-sectional study reflects AIIRD patients' acceptance and opinions about COVID-19 vaccination during the pandemic. However, people's attitudes and beliefs may change with time. Finally, our study looked at COVID-19 vaccination acceptance rates under the assumption that the vaccine was free; acceptability might be lower if the vaccine would come with out-of-pocket costs.

However, this study had several strengths; the questionnaire is reliable and straightforward. A face-to-face interview was conducted. The study also examined a wide range of demographic factors and health beliefs about COVID-19 vaccine. The study targeted an Egyptian cohort. To the best of our knowledge, this is the first paper that addresses the intention in Egyptian AIIRD patients towards COVID-19 vaccination.

Further studies on a large number of patients and from different rheumatology centers in Egypt are warranted to confirm these data and help improve the acceptancy of AIIRD patients towards COVID-19 vaccination. Multinational studies also are also needed to shed light on this topic.

In conclusion, the findings of this study are critical in determining the level of acceptancy and concerns about COVID-19 vaccination among AIIRD patients, as well as identifying motivators and barriers towards COVID-19 vaccination and, hence, recognizing effective strategies for increasing vaccine coverage in those populations including ongoing training and education. Contextual factors, such as media coverage of COVID-19, may influence vaccine beliefs and attitudes.

## Figures and Tables

**Table 1:** Demographic characteristics of the AIIRD population according to their decision regarding the COVID-19 vaccine (*n* = 206).

Variables, mean ± SD or *n* (%)	Total (*n* = 206)	COVID-19 vaccine acceptant group (*n* = 145)	COVID-19 vaccine hesitant group (*n* = 33)	COVID-19 vaccine resistant group (*n* = 28)	*P*
Age (years)	37.61 ± 10.67	37.45 ± 10.44	38.9 ± 12	36.43 ± 10.4	0.66
*Sex*					0.914
Female	173 (84)	122 (84.1)	27 (81.8)	24 (85.7)	
Male	33 (16)	23 (15.9)	6 (18.2)	4 (14.3)	
*Marital status*					0.294
Single	48 (23.3)	30 (20.7)	11 (33.3)	7 (25)	
Married	158 (76.7)	115 (79.3)	22 (66.7)	21 (75)	
*Occupation*					0.198
Employed	56 (27.2)	38 (26.2)	11 (33.3)	7 (25)	
Not employed	134 (65)	99 (69.3)	18 (45.5)	17 (60.7)	
Retired	3 (1.5)	3 (2.1)	0	0	
Not able to work due to disability	9 (4.4)	4 (2.8)	3 (9.1)	2 (7.1)	
Student	4 (1.9)	1 (0.7)	1 (3)	2 (7.1)	
*Residence*					0.877
Rural	89 (43.2)	61 (42.1)	15 (45.5)	13 (46.4)	
Urban	117 (56.8)	84 (57.9)	18 (54.5)	15 (53.6)	
*Education*					0.555
None	22 (10.7)	15 (10.3)	4 (12.1)	3 (10.7)	
Middle School	31 (15)	21 (14.5)	5 (15.2)	5 (17.9)	
High School	73 (35.4)	58 (40)	9 (27.3)	6 (21.4)	
College degree	63 (30.6)	41 (28.3)	13 (39.4)	9 (32.1)	
Postgraduate	17 (8.3)	10 (6.9)	2 (6.1)	5 (17.9)	
Active lifestyle	89 (43.2)	66 (45.5)	12 (36.4)	11 (39.3)	0.573
*Smoking habit*					0.463
Nonsmoker	187 (90.8)	131 (90.3)	29 (87.9)	27 (96.4)	
Former smoker	8 (3.9)	6 (4.1)	1 (3)	1 (3.6)	
Current smoker	11 (5.3)	8 (5.5)	3 (9.1)	0	
*Family income*					0.616
Not enough	34 (16.5)	17 (11.7)	10 (30.3)	7 (25)	
Enough but no saving	124 (60.2)	97 (66.9)	14 (42.4)	13 (46.4)	
Enough and saving	48 (23.3)	31 (21.4)	9 (27.3)	8 (28.6)	

^*∗*^ *p* < 0.05.

**Table 2:** Clinical and therapeutic data of the AIIRD population according to their decision regarding the COVID-19 vaccine (*n* = 206).

Variables, median (min-max) or *n* (%)	Total (*n* = 206)	COVID-19 vaccine acceptant group (*n* = 145)	COVID-19 vaccine hesitant group (*n* = 33)	COVID-19 vaccine resistant group (*n* = 28)	*P*
*AIIRD subtype*					0.749
SLE	85 (41.3)	61 (42.1)	12 (36.4)	12 (42.9)	
RA	54 (26.2)	38 (26.2)	7 (21.2)	9 (32.1)	
SSc	29 (14.1)	20 (13.8)	4 (12.1)	5 (17.9)	
BD	22 (10.7)	18 (12.4)	4 (12.1)	0	
Primary APLs	3 (1.5)	2 (1.4)	1 (3)	0	
AS	3 (1.5)	1 (0.7)	1 (3)	1 (3.6)	
DM	2 (1)	1 (0.7)	0	1 (3.6)	
PsA	1 (0.5)	1 (0.7)	0	0	
Vasculitis	1 (0.5)	1 (0.7)	0	0	
AOSD	1 (0.5)	1 (0.7)	0	0	
Overlap syndrome (SLE, RA)	5 (2.4)	1 (0.7)	4 (12.1)	0	
*Disease duration (years)*	5 (0.5–35)	5 (0.5–28)	5 (0.5–35)	8 (0.5–19	0.477
*Self-rated overall disease activity*	6 (0–10)	6 (0–10)	6 (0–10)	5 (0–10)	0.381
*Associated comorbidities*					
Diabetes mellitus	19 (9.2)	13 (9)	5 (15.2)	1 (3.6)	0.293
Hypertension	45 (21.8)	30 (20.7)	11 (33.3)	4 (14.3)	0.167
Chronic lung disease	18 (8.7)	13 (9)	3 (9.1)	2 (3.1)	0.95
Ischemic heart disease	2 (1)	2 (1.4)	0	0	0.655
Others	56 (27.2)	39 (26.9)	11 (33.3)	6 (21.4)	0.577
*History of COVID-19*	53 (25.7)	42 (29)	3 (9.1)	8 (28.6)	0.057
Hospitalization	8 (15.1)	6 (14.3)	1 (33.3)	1 (12.5)	0.629
ICU admission	3 (5.7)	2 (4.8)	1 (33.3)	0	0.079
*COVID-19 among relatives or friends*	84 (40.8)	55 (37.9)	16 (48.5)	13 (46.4)	0.478
Hospitalization	40 (19.4)	22 (15.2)	10 (30.3)	8 (28.6)	0.067
Death	23 (11.2)	14 (9.7)	6 (18.2)	3 (10.7)	0.389
*Therapeutic data*					
Corticosteroids	138 (67)	101 (69.7)	20 (60.6)	17 (60.7)	0.457
Hydroxychloroquine	94 (45.6)	64 (44.1)	19 (57.6)	11 (39.3)	0.291
Leflunomide	22 (10.7)	17 (11.7)	2 (6.1)	3 (10.7)	0.638
Methotrexate	66 (32)	50 (34.5)	7 (21.2)	9 (32.1)	0.339
Mycophenolate mofetil	42 (20.4)	29 (20)	7 (21.2)	6 (21.4)	0.977
Adalimumab	4 (1.9)	3 (2.1)	1 (3)	0	0.681
Infliximab	4 (1.9)	1 (0.7)	2 (6.1)	1 (3.6)	0.105
Etanercept	4 (1.9)	2 (1.4)	2 (6.1)	0	0.156
Golimumab	1 (0.5)	1 (0.7)	0	0	0.810
Tocilizumab	1 (0.5)	1 (0.7)	0	0	0.810
Secukinumab	1 (0.5)	1 (0.7)	0	0	0.810
Rituximab	2 (1)	1 (3)	1 (3.6)	0	0.09
Adherence to therapy	175 (85)	126 (86.9)	26 (78.8)	23 (82.1)	0.388

AOSD: adult-onset Still's disease, APLs: antiphospholipid syndrome, AS: ankylosing spondylitis, BD: Behcet's disease, DM: dermatomyositis, PsA: psoriatic arthritis, RA: rheumatoid arthritis, SLE: systemic lupus erythematosus, SSc: systemic sclerosis.

**Table 3:** Perception, beliefs, and attitudes of the AIIRD population towards COVID-19 and conventional and COVID‐19 vaccination (*n* = 206).

Statement, median (min-max) or *n* (%)	Total (*n* = 206)	COVID-19 vaccine acceptant group (*n* = 145)	COVID-19 vaccine hesitant group (*n* = 33)	COVID-19 vaccine resistant group (*n* = 28)	*P*
*COVID-19*					
Perception of higher risk of getting COVID-19 due to AIIRD	159 (77.2)	113 (77.9)	27 (81.8)	19 (97.9)	0.347
Perception of more severe COVID-19 due to AIIRD	172 (83.5)	124 (85.5)	27 (81.8)	21 (75)	0.237
Perception of higher risk of COVID-19 vaccine adverse events due to AIIRD	175 (75.2)	104 (71.7)	28 (84.8)	23 (82.1)	0.298
*Conventional vaccination (excluding COVID-19 vaccines)*					
Efficacy	7 (0–10)	7 (0–10)	3 (0–10)	3.5 (0–10)	<0.001^*∗*^
Security	7 (0–10)	7 (0–10)	5 (0–10)	4 (0–10)	<0.001^*∗*^
Usefulness	7 (0–10)	7 (0–10)	5 (0–10)	5 (0–10)	<0.001^*∗*^
Estimated knowledge	6 (0–10)	7 (0–10)	5 (0–10)	5 (0–10)	<0.001^*∗*^
*COVID-19 vaccination*					
COVID‐19 vaccine is important	163 (79.1)	130 (89.7)	21 (63.6)	12 (42.9)	<0.001^*∗*^
COVID‐19 vaccination to everyone in the community is important	158 (76.7)	131 (90.3)	18 (54.5)	9 (32.1)	<0.001^*∗*^
COVID‐19 vaccination should always be mandatory	139 (67.5)	119 (82.1)	13 (39.4)	7 (25)	<0.001^*∗*^
Concerns about COVID‐19 vaccination	154 (74.8)	128 (88.3)	16 (48.5)	10 (35.7)	0.703
COVID‐19 vaccination should always be compulsory for HCWs	170 (82.5)	132 (91)	21 (63.6)	17 (60.7)	<0.001^*∗*^
The vaccine's approval ensures its safety	130 (63.1)	113 (77.8)	12 (36.4)	5 (17.9)	<0.001^*∗*^
COVID‐19 vaccine may have adverse effects	156 (75.7)	108 (74.5)	27 (81.8)	21 (75)	0.703
COVID‐19 vaccine may be ineffective	141 (68.4)	95 (65.5)	25 (75.8)	21 (75)	0.412
An adverse reaction to a vaccine in the past	57 (27.7)	43 (29.7)	10 (30.3)	4 (14.3)	0.23
Against vaccination in general	47 (22.8)	28 (19.3)	11 (33.3)	8 (28.6)	0.174
Concerns for the acquisition of COVID‐19 from the vaccine	115 (55.8)	67 (46.2)	26 (78.8)	22 (78.6)	<0.001^*∗*^
I am not at a risk of developing complications if infected with COVID‐19	58 (28.2)	38 (26.2)	10 (30.3)	10 (35.7)	0.583
I am not at elevated risk to acquire COVID‐19	57 (27.7)	37 (25.5)	10 (30.3)	10 (35.7)	0.525
Vaccination is the best preventive measure for COVID‐19	101 (49)	84 (57.9)	12 (36.4)	5 (17.9)	<0.001^*∗*^

^*∗*^ *p* < 0.05. ARD: autoimmune rheumatic disease; HCWs: healthcare workers.

**Table 4:** List of types and adverse effects of COVID-19 vaccines in AIIRD patients who have received COVID-19 vaccine (*n* = 37).

Variables	Vaccinated AIIRD patients (*n* = 37), *n* (%)
*Type of COVID-19 vaccine received*	
Sinopharm	11 (29.7)
Astrazeneca	9 (24.3)
Pfizer	8 (21.6)
Sinovac	4 (10.8)
Moderna	1 (2.7)
Others	4 (10.8)
*Adverse effects of COVID-19 vaccines*	
Widespread muscle/joint pain	15 (40.5)
Fever or chills	12 (32.4)
Fatigue or sleepiness	6 (16.2)
Headache	6 (16.2)
Nausea	2 (5.4)
Vomiting	2 (5.4)
Chest pain-palpitations	2 (5.4)
Flare of the existing rheumatic disease	2 (5.4)
Rash	1 (2.7)
Poor appetite	1 (2.7)
Diarrhea	1 (2.7)
Mouth dryness	1 (2.7)
New rheumatic or other autoimmune disease	0
Anaphylaxis	0

ARD: autoimmune rheumatic disease.

## Data Availability

Data are available on request.

## Abbreviations

AIIRD: Autoimmune and autoinflammatory rheumatic disease
BD: Behçet's disease
COVID-19: Coronavirus disease 2019
RA: Rheumatoid arthritis
SARS-CoV-2: Severe acute respiratory syndrome coronavirus 2
SLE: Systemic lupus erythematosus
SSc: Systemic sclerosis
TV: Television
VA: Vaccine acceptant
VAXICOV study: VAccinations against COVID-19 study
VH: Vaccine hesitant
VR: Vaccine resistant
WHO: World Health Organization.
